# The efficacy of alendronate for the treatment of thalassemia-associated osteoporosis: a randomized controlled trial

**DOI:** 10.3389/fendo.2023.1178761

**Published:** 2023-05-10

**Authors:** Pokpong Piriyakhuntorn, Adisak Tantiworawit, Mattabhorn Phimphilai, Somdet Srichairatanakool, Waralee Teeyasoontranon, Thanawat Rattanathammethee, Sasinee Hantrakool, Chatree Chai-Adisaksopha, Ekarat Rattarittamrong, Lalita Norasetthada, Kanda Fanhchaksai, Pimlak Charoenkwan

**Affiliations:** ^1^Division of Hematology, Department of Internal Medicine, Faculty of Medicine, Chiang Mai University, Chiang Mai, Thailand; ^2^Division of Endocrinology and Metabolism, Department of Internal Medicine, Faculty of Medicine, Chiang Mai University, Chiang Mai, Thailand; ^3^Department of Biochemistry, Faculty of Medicine, Chiang Mai University, Chiang Mai, Thailand; ^4^Division of Nuclear Medicine, Department of Radiology, Faculty of Medicine, Chiang Mai University, Chiang Mai, Thailand; ^5^Division of Hematology and Oncology, Department of Pediatrics, Faculty of Medicine, Chiang Mai University, Chiang Mai, Thailand; ^6^Thalassemia and Hematology Center, Faculty of Medicine, Chiang Mai University, Chiang Mai, Thailand

**Keywords:** osteoporosis, bone mineral density, alendronate, bisphosphonate, CTX, P1NP, back pain, thalassemia

## Abstract

**Background:**

With adequate blood transfusion and iron chelation, thalassemia patients have a longer life expectancy and experience long-term metabolic complications, including osteoporosis, fractures, and bone pain. Alendronate, an oral bisphosphonate, is currently used to treat various types of osteoporosis. However, the efficacy for the treatment of thalassemia-associated osteoporosis remains unclear.

**Methods:**

We conducted a randomized controlled trial to evaluate the efficacy of alendronate for the treatment of osteoporosis in thalassemia patients. Patients were included if they were males (18–50 years) or premenopausal females with low bone mineral density (BMD) (Z-score < -2.0 SD) or positive vertebral deformities from vertebral fracture analysis (VFA). Stratified randomization was performed according to sex and transfusion status. Patients were 1:1 allocated to receive once weekly alendronate 70 mg orally or placebo for a total duration of 12 months. BMD and VFA were re-evaluated at 12 months. Markers of bone resorption (C-terminal crosslinking telopeptide of type I collagen; CTX) and bone formation (Procollagen type I N-terminal propeptide; P1NP), and pain scores were measured at baseline, 6 months, and 12 months. The primary outcome was the change of BMD. The secondary endpoints were changes in bone turnover markers (BTM) and pain scores.

**Results:**

A total of 51 patients received the study drug, 28 patients were assigned to receive alendronate and 23 patients to receive placebo. At 12 months, patients in the alendronate group had significant improvement of BMD at L1-L4 compared to their baseline (0.72 ± 0.11 vs 0.69 ± 0.11 g/cm^2^, p = 0.004), while there was no change in the placebo group (0.69 ± 0.09 vs 0.70 ± 0.06 g/cm^2^, p = 0.814). There was no significant change of BMD at femoral neck in both groups. Serum BTMs were significantly decreased among patients receiving alendronate at 6 and 12 months. The mean back pain score was significantly reduced compared to the baseline in both groups (p = 0.003). Side effects were rarely found and led to a discontinuation of the study drug in 1 patient (grade 3 fatigue).

**Conclusion:**

Alendronate 70 mg orally once weekly for 12 months effectively improves BMD at L-spine, reduces serum BTMs, and alleviates back pain in thalassemia patients with osteoporosis. The treatment was well tolerated and had a good safety profile.

## Introduction

Thalassemia is a common cause of inherited anemia in Thailand with estimated 3,000 new cases each year (1). Adequate blood transfusion and iron chelation lengthen the life span of thalassemia patients. As a result, these patients are experiencing long-term metabolic complications, including thalassemia bone diseases. Osteoporosis in thalassemia patients results from impaired bone remodeling process, which consists of decreased osteoblastic activity as well as increased osteoclastic function (2). The development of thalassemia-associated osteoporosis (TAO) is mainly influenced by bone marrow expansion, hypogonadism, imbalanced cytokine profiles, and a defective growth hormone-insulin-like growth factor-1 (GH-IGF-1) axis. In addition, iron overload, deferoxamine toxicity, vitamin D deficiency, and other endocrinopathies could be potential contributing factors (3, 4). The prevalence of TAO is as high as 50% in well-treated patients (5) and up to 70% in suboptimally-treated individuals (6). Fractures are frequently found among thalassemia patients at a young age in Thailand and worldwide (7, 8). To date, there is no consensus on the standard treatment of TAO. Previous studies have shown that bisphosphonates improve bone mineral density in thalassemia patients with osteoporosis due to an anti-bone resorptive effect (9–14).

Alendronate is an oral bisphosphonate which is convenient, safe, and widely available. Alendronate is currently used for the treatment of postmenopausal, glucocorticoid-induced, and male osteoporosis (15). The efficacy of alendronate for the treatment of TAO has been tested in a few previous studies, which were small, non-randomized, or retrospective approaches, and the results were inconsistent (11–14). Thus, we aimed to conduct a larger randomized controlled trial that included both transfusion-dependent (TDT) and non-transfusion-dependent thalassemia (NTDT) to assess the efficacy of alendronate in the setting of TAO.

## Materials and methods

### Study design and participants

We conducted a double-blind, randomized controlled trial to evaluate the efficacy of alendronate, compared with placebo, in patients with TAO. Eligible participants were ambulatory thalassemia patients aged 18–50 years (males) or more than 18 years (premenopausal females). Other hemoglobinopathies were allowed in case of compound heterozygosity with α- or β-thalassemia leading to clinically significant thalassemia syndrome. Patients had to be diagnosed with thalassemia-associated osteoporosis as defined by one of the following criteria, (i) a bone mineral density (BMD) Z-score of less than -2.0 SD at either the L1-L4 spine or the femoral neck on either side, measured by dual-energy X-ray absorptiometry (DXA; Hologic Discovery A, Hologic Company, Marlborough, MA, USA); or (ii) presence of vertebral deformity detected by vertebral fracture analysis (VFA; obtained in the supine position from T4 to L4 using the same machine as DXA). Key exclusion criteria were an estimated glomerular filtration rate of less than 30 ml per minute per 1.73 m^2^ of body-surface area, prior treatment with bisphosphonates, serum calcium below the lower normal limit, unable to stay in an upright position at least 30 minutes after taking the study drug, having uncorrected dental problems, or being pregnant. Informed consent was obtained from all patients.

### Procedures

Eligible patients were 1:1 randomized to receive either alendronate at a dose of 70 mg orally once weekly or a placebo in the morning on an empty stomach for 12 months. Computer-generated block randomization was performed according to sex and dependency of red cells transfusion (TDT or NTDT), with a block size of 4. The details of the randomization list were only known and kept by a department’s research officer. Calcium carbonate (with elemental calcium of 600 mg per day, taken after lunch or dinner) and vitamin D2 20,000 IU per week supplementations were given to all participants.

Patients were evaluated at baseline and every 3 months thereafter. At baseline, complete blood count and biochemical profile were measured, including calcium, phosphate, and vitamin D (as 25-hydroxy vitamin D) levels. Iron profile, in the form of serum ferritin, was measured. Patients were tested for chronic viral hepatitis B, hepatitis C, and human immunodeficiency virus infection. Tests for hypogonadism, hypothyroidism, abnormal parathyroid hormone, low insulin-like growth factor-1 (IGF-1) level, and diabetes mellitus were performed at baseline. Low IGF-1 was defined as less than the lower limit of normal IGF-1 level of that age and sex. Hormone replacement therapy was allowed during the study period.

### Outcomes

Two bone turnover markers (BTMs), a marker of bone resorption (C-terminal crosslinking telopeptide of type I collagen; CTX) and a marker of bone formation (Procollagen type I N-terminal propeptide; P1NP) were measured at baseline, 6 months, and 12 months. Pain scores at back and bone were evaluated using the numeric rating scale (0 point = no pain; 10 points = worst pain possible) at baseline, 6 months, and 12 months. Bone pain referred to generalized bone pain. DXA with VFA was re-evaluated at 12 months to determine the changes in BMD and degree of vertebral deformity.

The primary endpoint was the change of BMD at 12 months. Key secondary endpoints were the change of serum BTMs, back pain, and bone pain score at 12 months. The least significant change (LSC) was defined as more than 25% reduction of serum BTMs compared to baseline. The safety profile of the study drug was evaluated. Other outcomes were the prevalence of vertebral fracture and vitamin D deficiency among patients with TAO.

### Statistical analysis

We planned to recruit at least a total of 48 patients in order to detect the difference in mean BMD of 0.091 g/cm^2^ after 12 months of alendronate administration (12). Paired samples t-test was used to demonstrate the changes in BMD and BTMs after the study drug. Independent samples t-test and Chi-square test were used to evaluate differences between groups as appropriate. Wilcoxon signed-rank test was used to demonstrate the change of vertebral deformity severity in the same group. A marginal model with an unstructured correlation structure using generalized estimating equations (GEE) was used to evaluate the differences in pain scores at various time points after the study drug. A P-value of less than 0.05 was considered to indicate statistical significance. Statistical analysis was performed using Stata 16.1 (StataCorp LLC, USA).

The study was conducted with approval from the Institutional Research Ethics Committee at the Faculty of Medicine, Chiang Mai University (Ref. number 045/2018). This study was registered with the Thai Clinical Trial Registry, number TCTR20180219004. This study was supported by research grants from the Faculty of Medicine, Chiang Mai University (Grant number 026/2562), and the Thai Society of Hematology.

## Results

### Patients

**Figure 1:**
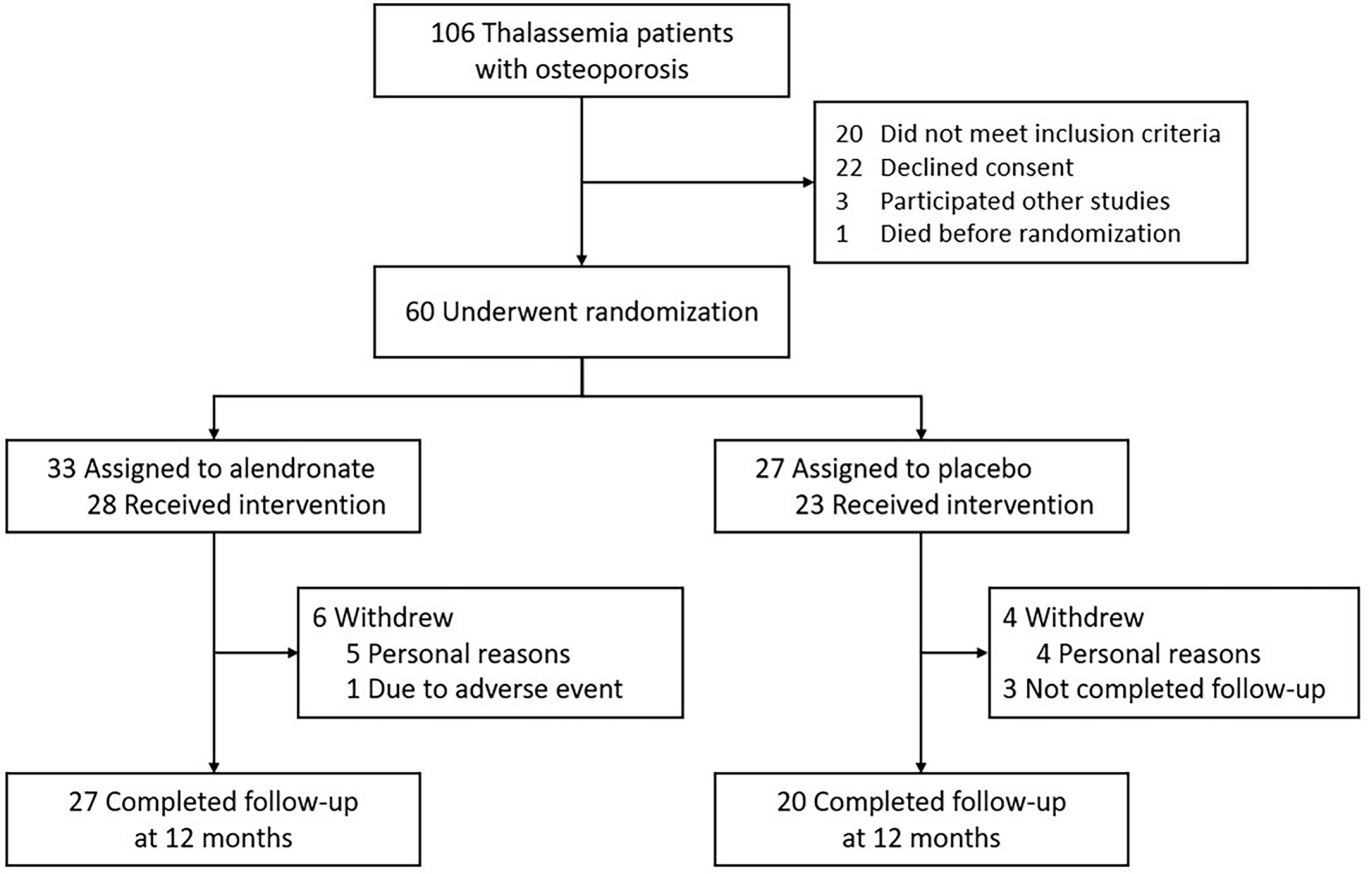
Study population flow.

From a total of 106 thalassemia patients with osteoporosis in the Chiang Mai University Hematology outpatient clinic between January 2018 and September 2018, 60 patients were eligible and underwent randomization. Thirty-three patients were assigned to receive alendronate, while 27 were assigned to receive placebo. Six and four patients from the alendronate and placebo groups withdrew their consent, respectively. At 12 months, 27 patients in the alendronate group and 20 patients in the placebo group completed the follow-up visit and were included in the analysis (**Figure 1**).

The baseline characteristics of patients were well-balanced (**Table 1**). The mean age of the patients was 29.1 ± 8.0 years. Most of the patients had beta-thalassemia diseases. Around two-thirds of patients had transfusion-dependent thalassemia, with mean units of transfused packed red blood cell of 1.66 units per month. The mean hemoglobin level was 7.2 ± 1.1 g/dL. Two-thirds of patients were splenectomized. Mean serum ferritin in both treatment groups were comparable. Eighty-six percent of patients had received iron chelation therapy.

**Table 1:** Patient characteristics.

Characteristic	Alendronate	Placebo
	(n = 28)	(n = 23)
Male sex, N (%)	12 (42.9)	12 (52.2)
Age, mean±SD	29.1±7.5	29.0±8.8
BMI (kg/m^2^), mean±SD	19.0±2.3	19.0±1.9
Current smoking, N (%)	2 (7.1)	1 (4.3)
Current alcohol use, N (%)	9 (32.1)	2 (8.7)
History of fracture, N (%)	12 (42.9)	8 (34.8)
Age at first fracture, median (range)	20 (3–28)	23.5 (7–30)
Parental hip fracture, N (%)	1 (3.6)	1 (4.3)
Transfusion dependent, N (%)	19 (67.9)	15 (65.2)
No. of transfused PRC (unit/month)	1.6±0.5	1.6±0.4
Thalassemia type, N (%)		
Homozygous β-thalassemia	8 (28.6)	9 (40.9)
β-Thalassemia/Hb E disease	18 (64.3)	12 (54.5)
Hb H disease	0	1 (4.5)
Hb H/Constant Spring disease	2 (7.1)	0
Splenectomized, N (%)	19 (67.9)	15 (65.2)
Current glucocorticoid use, N (%)	0	2 (8.7)
Received iron chelation therapy, N (%)	25 (89.3)	19 (82.6)
DFO	4 (14.3)	4 (17.4)
DFP	14 (50.0)	9 (39.1)
DFX	0	1 (4.3)
DFO + DFP	7 (25.0)	5 (21.7)
Hypogonadism, N (%)	5 (17.8)	7 (30.4)
Received HRT, N (%)	3 (60.0)	4 (57.1)
Hypothyroidism, N (%)	2 (7.1)	3 (13.0)
Subclinical hypothyroidism, N (%)	7 (25.0)	6 (26.1)
Diabetes mellitus, N (%)	2 (7.1)	1 (4.3)
Low IGF-1 level, N (%)	16 (57.1)	12 (52.2)
Vitamin D level (ng/ml), mean±SD	31.0±9.7	29.2±7.9
Normal (>30), N (%)	14 (50.0)	9 (39.1)
Insufficiency (20–30), N (%)	12 (42.9)	11 (47.8)
Deficiency (<20), N (%)	2 (7.1)	3 (13.0)
Viral profile, N (%)		
Chronic viral hepatitis B	2 (7.1)	1 (4.3)
Chronic viral hepatitis C	2 (7.1)	2 (8.7)
HIV infection	1 (3.6)	0
CBC parameters, mean±SD		
Hemoglobin level (g/dL)	7.4±1.2	6.9±0.8
WBC count (x10^9^/L)	13.5±5.4	13.1±6.0
Platelet count (x10^9^/L)	551.3±253.4	526.4±328.1
Nucleated RBC (/100 WBC), median (IQR)	95.5 (5.0, 249.3)	69.8 (4.3, 280.5)
Serum ferritin (μg/L), mean±SD	1,666±1,131	1,844±1,352
Serum bone turnover markers, mean±SD		
CTX (ng/ml)	0.56±0.50	0.46±0.23
P1NP (ng/ml)	82.99±71.47	72.05±53.36
BMD at L-spine (g/cm^2^), mean±SD	0.69±0.11	0.70±0.06
Z-score	-2.72±0.98	-2.55±0.65
BMD at femoral neck (g/cm^2^), mean±SD	0.66±0.09	0.65±0.06
Z-score	-1.50±0.72	-1.50±0.52
Vertebral deformity from VFA, N (%)	10 (35.7)	8 (34.8)
Mild	2 (20.0)	5 (62.5)
Moderate	6 (60.0)	2 (25.0)
Severe	2 (20.0)	1 (12.5)
Back pain, N (%)	10 (35.7)	10 (43.5)
Bone pain, N (%)	5 (17.9)	2 (8.7)

BMD, bone mineral density; BMI, body mass index; CBC, complete blood count; CTX, C-terminal crosslinking telopeptide of type I collagen; DFO, deferoxamine; DFP, deferiprone; DFX, deferasirox; HRT, hormone replacement therapy; IGF-1, insulin-like growth factor 1; IQR, interquartile range; P1NP, procollagen type I N-terminal propeptide; PRC, packed red cells; RBC, red blood cell; SD, standard deviation; VFA, vertebral fracture analysis; WBC, white blood cell.

The mean serum vitamin D level was 30.2 ± 8.9 ng/ml, and 54.9% of patients had low serum vitamin D. Patients in the placebo group tended to have more hypogonadism but not statistically significant (14.8% vs 30.4%, p = 0.18). Low IGF-1 level was common among thalassemia patients with osteoporosis (54.9%).

About 40% of patients had a history of trauma-related fracture, with a median age of 21 years (IQR 11 – 25) when they had the first fracture event. The prevalence of vertebral fractures among thalassemia patients with osteoporosis was 35.3%. Most patients were asymptomatic (11 of 18, 61.1%).

Prespecified analysis comparing the characteristics between TDT and NTDT patients revealed that the TDT group had more patients with low IGF-1 level (73.5% vs 17.6%, p <0.001) compared to the NTDT group. In addition, patients in the TDT group had higher mean serum ferritin level (2,122 ± 1,310 vs 994 ± 502 μg/L, p < 0.001). TDT patients tended to have more hypogonadism than NTDT group (29.4% vs 11.8%, p=0.161). However, the mean BMD Z-score, levels of BTMs, and history of fractures were not statistically different between groups.

### Efficacy

#### Bone mineral density and vertebral deformity

After 12 months of alendronate, there was a significant improvement in mean BMD at L1-L4 compared to baseline (0.72 ± 0.11 vs 0.69 ± 0.11 g/cm^2^, p = 0.004). However, there was no significant improvement in mean BMD at femoral neck after 12 months of the study drug. Patients in the placebo group had no significant change in BMD at both L1-L4 and femoral neck (**Table 2**). The mean percentage change of BMD at L1-L4 from baseline in the alendronate group was superior to the placebo group (+4.95 ± 8.04% vs -0.52 ± 9.93%, p = 0.042), while the difference was not observed at femoral neck (-0.63 ± 4.40% vs -0.62 ± 5.57%, p = 0.996). There was no new or increased vertebral deformity detected compared with their baseline in both groups. Also, there was no fracture observed among both groups of patients during the study follow-up period.

**Table 2:** Changes in bone mineral density at 12 months.

Site	Alendronate (n = 27)		P value	Placebo (n = 20)		P value
	Baseline	12 months		Baseline	12 months	
L1-L4						
BMD (g/cm^2^)	0.69±0.11	0.72±0.11	0.004	0.70±0.06	0.69±0.09	0.814
BMD Z-score	-2.76±0.99	-2.43±1.03	<0.001	-2.62±0.67	-2.55±0.74	0.601
BMC (g)	34.39±9.87	35.79±10.45	0.013	33.59±8.79	33.95±9.33	0.555
Femoral neck						
BMD (g/cm^2^)	0.65±0.08	0.64±0.08	0.502	0.64±0.06	0.64±0.05	0.51
BMD Z-score	-1.26±0.70	-1.25±0.72	0.778	-1.27±0.53	-1.31±0.52	0.571
BMC (g)	3.04±0.58	3.03±0.60	0.751	3.06±0.49	3.08±0.52	0.543

Data were presented as mean±SD; BMC, bone mineral content; BMD, bone mineral density.

#### Bone turnover markers

**Figure 2:**
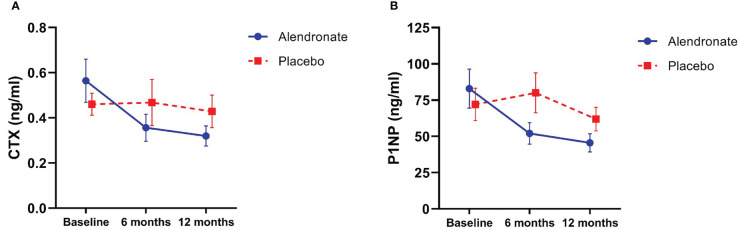
Changes in bone turnover markers; **(A)** C-terminal crosslinking telopeptide of type I collagen (CTX), and **(B)** procollagen type I N-terminal propeptide (P1NP).

After 12 months of the study drug, patients receiving alendronate had a significant reduction in serum CTX compared to baseline (0.32 ± 0.22 vs 0.57 ± 0.52 ng/ml, p = 0.002) (**Figure 2A**). Likewise, serum P1NP level showed a significant reduction at 12 months compared to baseline in the alendronate group (45.58 ± 31.37 vs 82.82 ± 73.99 ng/ml, p = 0.006) (**Figure 2B**). There was no significant change in BTMs in the placebo group. In addition, there was a significant proportion of participants who achieved LSC of serum CTX at 12 months in the alendronate group (72.0% vs 40.0%, p = 0.031). However, the proportion of participants who achieved LSC of serum P1NP was not significantly different between groups (72.0% vs 45.0%, p = 0.066).

#### Pain scores

**Figure 3:**
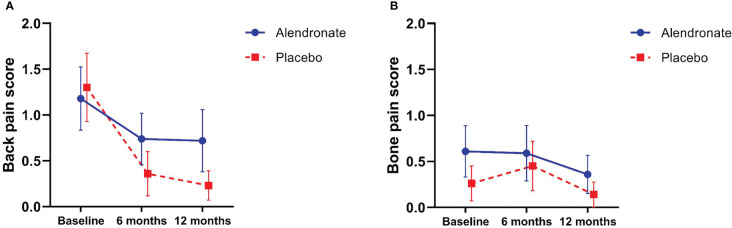
Changes in pain score; **(A)** Black pain score **(B)** Bone pain score.

The mean score of back pain was significantly reduced when compared to baseline in both groups (p = 0.003); however, there was no significant difference between groups (p = 0.222) (**Figure 3A**). Both alendronate and placebo did not significantly reduce bone pain among patients with TAO (p = 0.055) (**Figure 3B**). No non-steroidal anti-inflammatory drugs (NSAIDs) were prescribed to participants during the study.

### Safety

Side effects were rarely found. There was one patient who experienced grade 3 fatigue after administrating a single dose of alendronate, which led to drug discontinuation. No bisphosphonate-related adverse effects were found during the study period, including hypocalcemia, gastrointestinal side effects, osteonecrosis of the jaw, or atypical femur fracture.

## Discussion

Currently, bisphosphonates are the mainstay of treatment of TAO, particularly the intravenous forms (8, 16, 17). However, the efficacy of oral-form alendronate in increasing BMD in thalassemia is still controversial (11–14), as also the lack of evidence on the alleviation of back and bone pain. Thus, we decided to conduct a randomized, placebo-controlled study to evaluate the efficacy of alendronate for the treatment of TAO in terms of improvement of BMD together with back and bone pain reduction.

Alendronate 10 mg once daily for 24 months has been shown to improve BMD only at femoral neck in 9 beta-thalassemia patients with hypogonadism who received hormone replacement therapy (HRT) (11). Since hypogonadism predominantly affects BMD at L-spine (18–20), it may attenuate the effect of alendronate at L-spine when patients receive HRT. Our study demonstrated that alendronate improved only BMD at L-spine, which was possibly due to the low prevalence of hypogonadism (23.5%) compared to prior study. Likewise, a large retrospective study in 218 thalassemic patients revealed that once-weekly alendronate 70 mg for 1 year improved BMD only at L-spine (13). This finding supports the result of our randomized study. In another single-arm study including 96 thalassemic patients, alendronate for 12 months has been demonstrated efficacy in improving BMD at both L-spine and femoral neck (12). The proportion of TDT and NTDT in this study was comparable with our current study. Since the magnitude of BMD improvement was more evident in L-spine, the efficacy of alendronate for improving BMD at L-spine was again affirmed. Even once-weekly 70 mg of alendronate failed to demonstrate the improvement of BMD at lumbar spine and femoral neck in 29 Greek Cypriot thalassemia major patients, there was a trend toward improvement of BMD at L-spine (mean T-score -2.720 to -2.602, p = 0.059) compared to the femoral neck (mean T-score -2.035 to -2.007, p = 0.829) (14). The possible explanation could be that transfusion-dependent patients may have a more impaired bone remodeling process that leads to a blunt response to alendronate.

In addition to mean BMD improvement, fewer patients in the alendronate group had declined BMD at L1-L4 from their baseline (33.3% vs 50%). This suggests that although alendronate did not improve BMD in all patients, it helped slow the rate of BMD reduction. Since one-third of patients did not adequately respond to 12 months of alendronate, the longer treatment duration, switching to more potent anti-resorptive agents such as zoledronate (10, 13) or denosumab (21), or switching to another class of anti-osteoporotic treatment such as teriparatide (22), or strontium ranelate (23) should be considered in these patients.

The efficacy of alendronate in the reduction of the risk of fracture has been shown regardless of the improvement in BMD among postmenopausal women (24). Previous studies found that P1NP and CTX assays have good precision and low analytical coefficient of variation in thalassemia patients with osteoporosis (25, 26). Subsequent analysis of the Fracture Intervention Trial (FIT) revealed that reductions of BTMs were associated with bone fracture reduction regardless of the improvement in BMD in osteoporotic postmenopausal women treated with alendronate (27). Therefore, the decrease of BTMs may be another surrogate for bone fracture in addition to increasing bone density.

LSC was defined as the least significant change in bone marker level that results in clinical significance. It has been proposed that the LSC was approximately 25% for serum CTX and P1NP levels (28). Moreover, in the FIT study (27), a decrease in serum P1NP of more than 30% was associated with a 55% less vertebral fracture. In our study, the use of alendronate significantly reduced serum CTX and P1NP with mean percent changes of 33.2% and 37.1%, respectively. In addition, 72% of patients receiving alendronate achieved LSC of serum BTMs compared with only 40% in the placebo group. These findings support that alendronate is effective in increasing BMD at L-spine, reducing serum BTMs, and potentially decreasing vertebral fracture risk in thalassemia patients.

The most commonly reported adverse event of alendronate is upper gastrointestinal tract side effects found in 41–47% among postmenopausal osteoporotic patients (29, 30). However, the incidences were not different from the placebo group, which could be explained by the high rate (75%) of NSAIDs use in both groups. Adverse effects of alendronate were rarely found in our study. Any gastrointestinal side effects were not found in this current study. This could be because our participants were instructed to drink at least 200 ml of water and stay upright for at least 30 minutes after taking the study drug. Also, no NSAIDs were prescribed to our participants during the study period. Only one patient (3.5%) experienced grade 3 fatigue after taking a single dose of alendronate. His symptom spontaneously improved in the next 24 hours without seeking medical care. Hence, we infer that alendronate is relatively safe and well-tolerable.

Another important endpoint is back pain, which may affect patients’ quality of life. In previous studies, zoledronate significantly reduced bone pain in thalassemia with osteoporosis (10, 31), while neridronate led to a meaningful reduction in back pain (9). Our study showed that alendronate significantly reduced back pain in thalassemia with osteoporosis, while bone pain did not significantly change after the study drug. The explanation could be the low prevalence (13.7%) and low severity of bone pain in the study population, resulting in the difficulty in demonstrating the effect of alendronate on bone pain reduction.

Not all thalassemia patients with low BMD need pharmacological treatment. According to The ISCD 2013 Pediatric Official Positions (32), osteoporosis diagnosis should not depend only on BMD. At least one vertebral compression fracture (a loss of vertebral height of over 20%), without evidence of local disease or high-energy trauma, suggests the diagnosis of osteoporosis. In the absence of vertebral fractures, the diagnosis of osteoporosis can be made if there is both a clinically significant fracture history and a BMD Z-score below -2.0. Clinically significant fracture history is defined as two or more long bone fractures by age 10, or three or more long bone fractures at any age up to 19. According to international guidelines, patients with established osteoporosis should be given medical treatment for fracture prevention (33, 34). In addition, pharmacological treatment for patients without history of significant fracture who had progressively declined BMD despite non-pharmacological interventions, including adequate blood transfusion with iron chelation, exercise, calcium and vitamin D supplementation, hormonal replacement, should be considered.

There were some limitations of this study. First, since the follow-up period is relatively short, it was unable to measure fracture rate as an important clinical outcome. Therefore, we also measured BTMs, which may correlate with fractures among patients receiving alendronate from the previous report. In addition, VFA after the study drug was also performed and no new vertebral fracture was found during the study period. Second, the number of patients in the study was relatively small. However, we recruited more than the expected sample size, and there was a low rate of incomplete follow-up (7.8%). Last, we did not collect the data on diet and physical activity of the participants which may affect the study outcomes. However, we prescribed calcium and vitamin D supplements to all participants to ensure the adequacy of these daily requirements while taking study drug.

## Conclusion

Alendronate 70 mg orally once weekly for 12 months is effective in improving BMD at L-spine, reducing serum BTMs, and alleviating back pain in thalassemia patients with osteoporosis. The treatment was well tolerated and had a rather good safety profile.

## Data availability statement

The data analyzed in this study is subject to the following licenses/restrictions: The data that support the findings of this study are available from the corresponding author, AT, adisak.tan@cmu.ac.th upon reasonable request. The data are not publicly available due to privacy or ethical restrictions. Requests to access these datasets should be directed to adisak.tan@cmu.ac.th.

## Ethics statement

The studies involving human participants were reviewed and approved by the Institutional Research Ethics Committee at the Faculty of Medicine, Chiang Mai University (Ref. number 045/2018). The patients/participants provided their written informed consent to participate in this study.

## Author contributions

Conceptualization, PP and AT. Methodology, PP, MP, WT and SS. Validation, PP and AT. Formal Analysis, PP and AT. Investigation, PP and AT. Data Curation, PP. Writing-Original Draft Preparation, PP and AT. Writing-Review & Editing, TR, SH, CC, ER, LN, KF, PC. Supervision, AT. Funding Acquisition, AT. All authors contributed to the article and approved the submitted version.

## Glossary

**Table:**

BMD	bone mineral density
BTMs	bone turnover markers
CBC	complete blood count
CTX	C-terminal crosslinking telopeptide of type I collagen
DFO	deferoxamine
DFP	deferiprone
DFX	deferasirox
DXA	dual-energy X-ray absorptiometry
FIT	Fracture Intervention Trial
GEE	generalized estimating equations
HRT	hormone replacement therapy
IGF-1	insulin-like growth factor-1
IQR	interquartile range
LSC	least significant change
NSAIDs	non-steroidal anti-inflammatory drugs
NTDT	non-transfusion-dependent thalassemia
P1NP	procollagen type I N-terminal propeptide
PRC	packed red cells
RBC	red blood cell
SD	standard deviation
TAO	thalassemia-associated osteoporosis
TDT	transfusion-dependent
VFA	vertebral fracture analysis
WBC	white blood cell
